# “A small leak will sink a great ship”: hypoxia-inducible factor and group III pulmonary hypertension

**DOI:** 10.14800/rci.1213

**Published:** 2016-03-14

**Authors:** Andrew J. Bryant, Edward W. Scott

**Affiliations:** 1 Department of Medicine, Division of Pulmonary, Critical Care and Sleep Medicine, University of Florida College of Medicine, Gainesville, FL 32610-0225, USA; 2 Department of Molecular Genetics & Microbiology, University of Florida College of Medicine, Gainesville, FL 32610-0225, USA

**Keywords:** Hypoxia-inducible factor (HIF), pulmonary hypertension, CCN, vascular permeability

## Abstract

Pulmonary hypertension complicating idiopathic pulmonary fibrosis, also known as secondary pulmonary hypertension, represents a major source of morbidity and mortality in affected patients. While the study of primary pulmonary arterial hypertension has yielded several therapies, the same is not true for the treatment of pulmonary hypertension secondary to pulmonary fibrosis. Recent studies have indicated an important role of hypoxia-inducible factor (HIF) – a regulatory protein that is vital in adaptation to hypoxic conditions – in the development of secondary pulmonary hypertension. HIF influences development of hypoxia-induced pulmonary hypertension through alteration in voltage-gated potassium channels and homeostatic calcium regulation, resulting in disruption of endothelial cell-cell communication, and eventual vascular remodeling. This article summarizes salient literature related to HIF and secondary pulmonary hypertension, in addition to proposing a final common pathway in known mechanistic pathways that result in endothelial barrier integrity loss – vascular “leak” – primarily through a shared endothelial-epithelial signaling protein family, CCN.

## Introduction

Idiopathic pulmonary fibrosis (IPF) is a progressive parenchymal lung disease without significant disease-modifying treatment. Survival time after diagnosis is widely variable and depends on a variety of factors including age, pulmonary function testing and degree of fibrosis. One additional factor contributing to death associated with IPF is the development of secondary pulmonary hypertension, defined as WHO group III pulmonary hypertension – associated with lung diseases and/or hypoxia. Prevalence of secondary pulmonary hypertension in IPF ranges from 32% to 85%, and importantly is associated with worse exercise capacity, quality of life and survival compared to patients with IPF and no evidence of pulmonary hypertension ^[1]^. Unfortunately, the available medical therapies for treatment of primary pulmonary arterial hypertension are either ineffective in the treatment of secondary pulmonary hypertension ^[2]^, or provide only a small potential benefit in secondary trial outcomes ^[3]^. To this end, there is a large need for novel therapies based on alternative mechanistic pathways in disease development.

One such candidate pathway is that of hypoxic signaling in the pathogenesis of secondary pulmonary hypertension, specifically the role of hypoxia-inducible factor (HIF). HIF is a key regulator of cellular adaptation to hypoxia, leading to increased transcription of hypoxia-response element genes, such as vascular endothelial growth factor (VEGF) and platelet-derived growth factor (PDGF). Existing in nature as a heterodimer with an α- and β-subunit, two isoforms of HIF (HIF1α and HIF2α) have been shown to influence development of secondary pulmonary hypertension in a chronic hypoxia model of secondary pulmonary hypertension ^[4, 5]^. While the literature on the topic is sparse, at least one murine study utilizing a model of pulmonary fibrosis – adenoviral delivery of TGF-β1 – showed that administration of a downstream hypoxia-response element target protein (VEGF) ameliorated pulmonary hypertension, yet may have exacerbated the fibrotic response ^[6]^.

The purpose of this article is to both explore current models of how HIF influences development of secondary pulmonary hypertension, and to examine potential future directions in research and treatment of this prevalent – conservatively, up to 4 per 100,000 people in the United States – and devastating illness ^[7]^.

## HIF and calctum homeostasis

Examining the effect of calcium antagonists on hypoxic pulmonary vasoconstriction, it is known that the hypoxic mechanism is critically dependent on the transmembrane influx of calcium ^[8]^. It has also been known for some time that hypoxia acts directly on the pulmonary artery smooth muscle to alter Ca^2+^ transport and increase contractility ^[9]^. More recently, in a model of chronically hypoxic rats, endothelin-1 (ET-1) – a potent vasoactive peptide in pulmonary vasculature – was demonstrated to increase the calcium sensitivity of pulmonary artery smooth muscle cells, suggesting a shared downstream mediator with pulmonary arterial hypertension ^[10]^.

Investigators from The Johns Hopkins University have been integral in establishing a link between HIF and calcium channel homeostasis in the development of secondary pulmonary hypertension. In one study, the group exposed mice to three weeks of 10% FiO_2_ and treated with either a HIF-1 translational inhibitor or vehicle. They were then able to demonstrate that not only did HIF-inhibition blunt the increase in right ventricular pressure and remodeling, it did so – in part – by decreasing the amount of intracellular calcium within pulmonary artery smooth muscle cells ^[11]^. This was independent of whether HIF-inhibition occurred prior to or after development of hypoxia-induced pulmonary hypertension.

While tonic hypoxic vasoconstriction almost certainly plays a role in the long-term remodeling of the pulmonary vasculature, it does not by itself establish a link with the late manifestations of disease demonstrating perivascular fibrosis and vessel thickening. To this end, in a second related study, the same group established a decrease in pulmonary artery smooth muscle cell migration with inhibition of hypoxia-induced, and calcium channel-mediated, development of pulmonary hypertension ^[12]^, suggesting a potential mechanism for a chronic HIF-controlled change in vascular remodeling.

## HIF and potassium channel regulation

A novel potassium channelopathy, KCNK3, was recently linked to the development of familial pulmonary hypertension, suggesting a potential role of potassium channels in the development of secondary pulmonary hypertension ^[13]^. A key determinant of intracellular calcium concentration in the pulmonary artery smooth muscle cell is the resting membrane potential, as this influences voltage dependent sarcolemmal Ca^2+^ channels. The resting membrane potential is in turn tightly regulated by voltage-gated potassium channels. These channels are known to have impaired function within the cell membrane upon exposure to chronic hypoxia. In a study exposing isolated rat intrapulmonary arterial cells to greater than 24 hours of hypoxia, Wang and colleagues ^[14]^ showed that the decrease in K_V_ channel subtypes 1.2 and 1.5 was a transcriptionally regulated event, data that were later confirmed in an in vivo model of subacute hypoxic exposure with subsequent development of secondary pulmonary hypertension ^[15]^. The phenotype is rescued with gene therapy targeted to increase K_V_1.5 channel expression, with decrease in hypoxic vasoconstriction and resolution of pulmonary hypertension ^[16]^.

The link between hypoxia exposure and subsequent alteration in K_V_ channel expression was later found to be at least partially HIF-mediated by Bonnet and colleagues. In their study, a rodent model of pulmonary hypertension with activation of HIF1α had inhibited expression of voltage-gated channel, K_V_1.5 ^[17]^. Blocking HIF activation decreased pulmonary vasoconstriction and downstream vascular remodeling, suggesting that manipulation of the protein pathway may lead to further pharmaceutical targets of disease.

## Hypoxia, endothelial cell communication and vascular integrity

A clue linking the mechanism of the above derangements with the subsequent development of secondary pulmonary hypertension can be found in the examination of BMPRII-related pulmonary arterial hypertension. It has previously been shown that BMP receptor signaling controls vascular remodeling during angiogenesis by maintaining the expression of endothelial regulatory molecules, with the attenuation of the signal resulting in predisposition to compromised vascular integrity ^[18]^. Using a model of pulmonary hypertension associated with pulmonary fibrosis, our group has recently demonstrated that mice with BMPRII mutation expressed a high amount of HIF1α in the lung, corresponding with worsening pulmonary hypertension ^[19]^. Thus, vascular “leak”, related to hypoxic signaling, may play a role in the progression of pulmonary hypertension. We also know that calcium entry into the endothelium influences barrier integrity, leading to immediate and delayed clinical consequences such as alveolar flooding and impairment of gas exchange ^[20]^. Prior work by Irwin and colleagues ^[21]^ demonstrated that not only does hypoxic-exposure in cultured human pulmonary artery endothelial cells cause increased albumin permeability and HIF1α accumulation, but treatment of hypoxia-exposed cells with HIF1α siRNA lowers monolayer permeability. A similarly designed study examining hypoxic exposure in an in vivo murine model found the same results, with downstream inhibition of HIF-dependent molecules resulting in protection against extravasation of Evans blue dye, indicating preserved barrier integrity ^[22]^.

Consequently, targeting a HIF-mediated molecular pathway that regulates both intracellular calcium and endothelial cell communication – influencing vascular integrity – would logically be of potential therapeutic benefit. The CCN family (primarily Cyr61, CTGF and NOV) are one such group of regulatory proteins that are involved in both endothelial cellular signaling and intracellular calcium trafficking ^[23]^, and therefore represent an attractive area of research in the treatment of secondary pulmonary hypertension. CCN proteins are tightly regulated by HIF under hypoxic conditions involved in a diverse array of tissue beds and functions, such as appropriate placental invasion in trophoblast cell lines (Cyr61 and NOV) ^[24]^, chondrocyte development and survival (CTGF) ^[25]^ and gastric cancer cell invasion (Cyr61) ^[26]^. In addition CCN-4, also known as WISP-1, has been shown to be necessary in the development of alveolar epithelial cell hyperplasia and epithelial-to-mesenchymal transition (EMT) leading to pulmonary fibrosis ^[27]^. Such a unique array of functions provides an interesting intersection between epithelial and endothelial cell biology, making the CCN pathway a very attractive target for future research ^[28, 29]^. In support of this assertion, our lab has recently shown that through deletion of HIF signaling within the vascular endothelium of mice, pulmonary hypertension is normalized in both the pulmonary fibrosis and chronic hypoxia models of disease ^[30]^. We furthermore demonstrated a correlative relationship with decreased CTGF expression, an alteration in vascular permeability, and degree of vascular remodeling. These findings support further exploration into tissue-specific targeting of the CCN family of proteins, including CTGF, for pharmacologic manipulation of the hypoxia-signaling pathway.

## Conclusions

While much is known from existing literature on the development of secondary, mainly hypoxia-induced, pulmonary hypertension, this knowledge base has afforded little in the way of directed therapy. The mechanism that HIF plays in the development and progression of disease has provided intriguing insight into the future of treatment. Working on the background of established pathways, including the role of HIF in potassium-channel mediated cell membrane potential and calcium homeostasis, a new direction has been established for examining shared pathways in disruption of the barrier integrity of the lung. Pursuing research along these lines, with the CCN family of proteins being a primary candidate for study, will provide the impetus to follow Benjamin Franklin’s timeless advice, and avoid allowing a “small leak” to continue to plague our efforts at preserving our pulmonary vessel, a “great ship.”

## Figures and Tables

**Figure 1:**
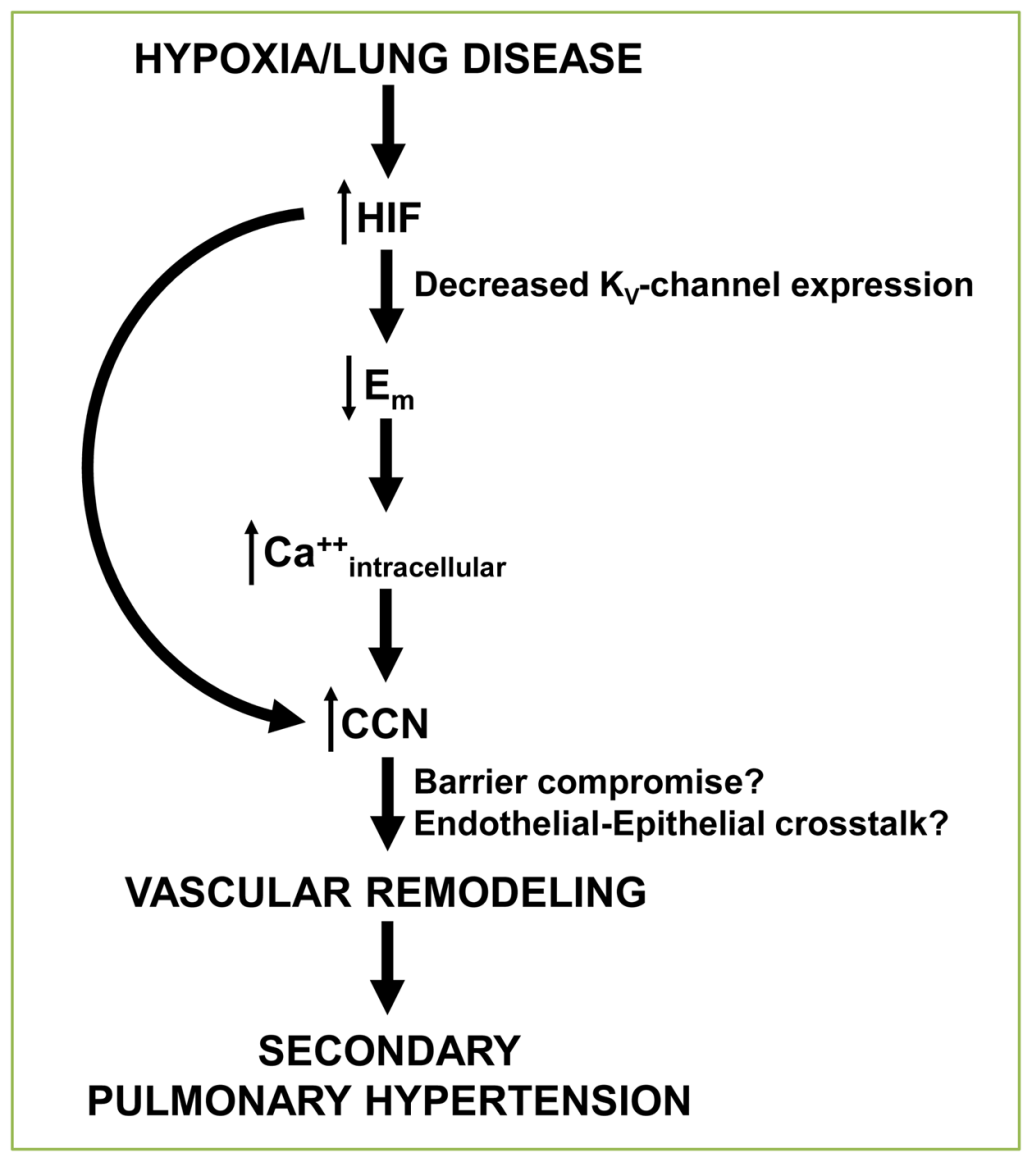
Proposed mechanism of HIF-mediated development of secondary pulmonary hypertension.
